# Dorsal and Ventral Stream Function in Children With Developmental Coordination Disorder

**DOI:** 10.3389/fnhum.2021.703217

**Published:** 2021-11-24

**Authors:** Serena Micheletti, Fleur Corbett, Janette Atkinson, Oliver Braddick, Paola Mattei, Jessica Galli, Stefano Calza, Elisa Fazzi

**Affiliations:** ^1^Unit of Child Neurology and Psychiatry, ASST Spedali Civili of Brescia, Brescia, Italy; ^2^Faculty of Brain Sciences, University College London, London, United Kingdom; ^3^Department of Experimental Psychology, University of Oxford, Oxford, United Kingdom; ^4^Department of Clinical and Experimental Sciences, University of Brescia, Brescia, Italy; ^5^Unit of Biostatistics and Bioinformatics, Department of Molecular and Translational Medicine, University of Brescia, Brescia, Italy

**Keywords:** developmental coordination disorder, motion sensitivity, form sensitivity, dorsal stream, ventral stream

## Abstract

Dorsal stream cortical networks underpin a cluster of visuomotor, visuospatial, and visual attention functions. Sensitivity to global coherence of motion and static form is considered a signature of visual cortical processing in the dorsal stream (motion) relative to the ventral stream (form). Poorer sensitivity to global motion compared to global static form has been found across a diverse range of neurodevelopmental disorders, suggesting a “dorsal stream vulnerability.” However, previous studies of global coherence sensitivity in Developmental Coordination Disorder (DCD) have shown conflicting findings. We examined two groups totalling 102 children with DCD (age 5–12 years), using the “Ball in the Grass” psychophysical test to compare sensitivity to global motion and global static form. Motor impairment was measured using the Movement-ABC (M-ABC). Global coherence sensitivity was compared with a typically developing control group (*N* = 69) in the same age range. Children with DCD showed impaired sensitivity to global motion (*p* = 0.002), but not global form (*p* = 0.695), compared to controls. Within the DCD group, motor impairment showed a significant linear relationship with global form sensitivity (*p* < 0.001). There was also a significant quadratic relationship between motor impairment and global motion sensitivity (*p* = 0.046), where poorer global motion sensitivity was only apparent with greater motor impairment. We suggest that two distinct visually related components, associated with global form and global motion sensitivity, contribute to DCD differentially over the range of severity of the disorder. Possible neural circuitry underlying these relationships is discussed.

## Introduction

Measures of coherence sensitivity to global visual form and global visual motion have been proposed as indicators of functioning in the ventral and dorsal cortical streams, respectively (Atkinson et al., 1997; Gunn et al., 2002; Braddick et al., 2003; Atkinson, 2017a). In typically developing infants and children these measures have been used to define the developmental trajectory of sensitivity to global visual motion and global visual form (Gunn et al., 2002; Atkinson and Braddick, 2005; Braddick et al., 2016). The maturation of global visual motion sensitivity is both delayed and more variable than that found for the maturation of global visual form sensitivity across a diverse range of neurodevelopmental disorders (Atkinson, 2017b), such as Developmental Dyslexia (e.g., Hansen et al., 2001; Conlon et al., 2009), Autism Spectrum Disorders (e.g., Spencer et al., 2000; Pellicano et al., 2005; Milne et al., 2006), Williams syndrome (e.g., Atkinson et al., 1997, 2006; Atkinson, in press), Fragile X syndrome (e.g., Kogan et al., 2004), and also prematurity (e.g., Guzzetta et al., 2009; Taylor et al., 2009), and hemiplegia (e.g., Gunn et al., 2002). These findings of greater deficits in sensitivity to global motion compared to global static form have led to the concept of “dorsal stream vulnerability,” a cluster of deficits in not only global visual motion perception but also visuomotor actions and visual attention (Braddick et al., 2003; Atkinson and Braddick, 2011; Atkinson, 2017b). The present study examined the concept of dorsal stream vulnerability in children with Developmental Coordination Disorder (DCD).

DCD is a neurodevelopmental disorder characterised by a primary motor impairment affecting up to 5–6% of children (Blank et al., 2019). It is marked by clumsiness, lack of coordination, and poor balance, which negatively and persistently affect activities of daily living (American Psychiatric Association (APA), 2013). The aetiology of DCD is currently thought to be multifactorial as no single cause has been identified; both genetic and environmental influences have been implicated (Gomez and Sirigu, 2015). The prevalence of DCD is higher in males than females, with estimates of two to three males for every female diagnosed (Lingam et al., 2009; Faebo Larsen et al., 2013). For a diagnosis of DCD to be made, symptoms must be present in early childhood and motor impairments must be in excess of those associated with any intellectual disability (American Psychiatric Association (APA), 2013). The Movement-ABC (M-ABC; Henderson and Sugden, 1992; Henderson et al., 2007) is the most commonly used test battery for the assessment of motor impairment in DCD (Blank et al., 2019).

Factors that may contribute to DCD symptomatology have been examined in meta-analyses conducted by Wilson and McKenzie (1998) and Wilson et al. (2013). Wilson et al. (2013) reported large mean effect sizes (d_w_ > 1.2) for visual perceptual tasks and complex visuospatial tasks involving motor components. Visuomotor coordination is one of the primary functions of the dorsal stream, alongside visuospatial skills and the control of attention (Atkinson, 2000, 2017b; Kravitz et al., 2011). Dorsal stream processing of global visual motion provides cues necessary for detecting direction of heading and for accurate locomotion, postural control and gross motor skills (Burr et al., 1998, 2001; Gepner and Mestre, 2002). Indeed, two recent studies have shown that global motion coherence sensitivity is related to visuomotor performance in children (Braddick et al., 2016; Chakraborty et al., 2017). Given that visuomotor coordination is one of the primary functions of the dorsal stream, it might be expected that children with DCD would share with other neurodevelopmental disorders poor sensitivity to motion coherence relative to form coherence. However, previous studies that have specifically explored global form and global motion sensitivity in DCD, using a matched groups approach, have given inconclusive results (Johnston et al., 2017).

O’Brien et al. (2002) reported that whilst global translational motion sensitivity was slightly better for eight 7–11 year olds with developmental dyspraxia compared to 50 controls, global form sensitivity was poorer. Sigmundsson et al. (2003) reported that 13 “clumsy” 10 year olds demonstrated both poorer global form and global translational motion sensitivity than typical controls. However, Sigmundsson et al. (2003) did not explicitly test the interaction between group and coherence sensitivity to form and motion to examine whether motion sensitivity was significantly lower than form sensitivity, relative to controls. The relevance of the results of O’Brien et al. (2002) and Sigmundsson et al. (2003) to DCD is difficult to assess as participants had not received a formal diagnosis of DCD and in the Sigmundsson et al. (2003) study, were allocated to groups by their performance on the M-ABC alone.

In a comparison of eleven 6–12 year olds with a diagnosis of DCD and controls, Wilmut and Wann (2008) reported that global concentric form and rotational motion sensitivity did not differ between groups. Purcell et al. (2012) compared radial (looming) motion sensitivity in eleven 6–11 year olds with DCD to controls, reporting that children with DCD showed a marked impairment in sensitivity to radial motion speed. This impairment was particularly evident when the moving object was not fixated within central vision, but no comparative measurement of form sensitivity was obtained.

These previous studies examining global form and motion sensitivity in DCD have involved only small numbers of participants (*N* = 8–13) with differing criteria for inclusion and exclusion as cases of DCD. Furthermore, in many of these studies different stimuli and tasks were used for comparing global form and motion sensitivity (Johnston et al., 2017). Sigmundsson and Haga (2010) recommend all DSM criteria to be checked, alongside the assessment of motor competency, in order for a diagnosis of DCD to be made.

The present study aimed to examine whether children with DCD show dorsal stream vulnerability as indexed by a selective impairment in global motion coherence sensitivity, compared to global form coherence sensitivity. A much larger sample (*N* > 100) of children with DCD was tested than in earlier reports, recruited in two studies, one in London, United Kingdom and one in Brescia, Italy. Children with DCD in both studies met defined DSM-5 criteria for DCD, which were validated by their scores on the M-ABC. Global form and motion sensitivity were assessed with the “Ball in the Grass” test, which is suitable for children as young as 4 years and for which extensive normative data exist (Atkinson and Braddick, 2005; Braddick et al., 2016). The size and age range (5–12 years) of the DCD group made it possible to examine (a) whether there is any association of global form and motion coherence sensitivity with individuals’ level of motor deficit as reflected in M-ABC scores; (b) whether this relationship is age-dependent; (c) whether the relationship is influenced by overall cognitive ability.

## Materials and Methods

### Ethical Approval

Full ethical approval was granted for the London study by the UCL ethics committee (2807/002) and for the Brescia study by the ethics committee of Brescia (NP 3513). Before advertising the research to individuals with DCD in the United Kingdom, the research was granted additional approval from the Dyspraxia Foundation Ethics Committee.

### Participants

#### Children With Developmental Coordination Disorder (Developmental Coordination Disorder Group)

Children with a diagnosis of DCD were recruited in London (London Group *N* = 17) and Brescia, Italy (Brescia Group *N* = 85). The enrolled children had to meet the following criteria: English/Italian native speakers, aged 5:0–12:11 years old, good binocular visual acuity (≥0.8) to easily detect the stimuli, with performance on the M-ABC lower or equal to the 15th percentile. All children had either normal vision or no history of visual problems beyond corrected refractive errors and had no prior experience in visual psychophysics testing. Participation was voluntary, and children with DCD and their caregivers were reimbursed for their travel expenses in the London study. Details of the groups are presented in Table 1.

**TABLE 1:** Demographic and clinical data of the samples.

**Groups**	**N (M/F)**	**Age (years) Mean (SD)**	**Cognitive assessments Standard scores Mean (SD)**				**Additional diagnoses and clinical data**						**Movement ABC Scaled scores Mean (SD)**			
			**BPVS-II**	**CPM**	**WPPSI III-WISC IV (Verbal IQ)**	**WPPSI III-WISC IV (Full IQ)**	**ADHD**	**Specific learning disorder**	**Speech and language disorder**	**Autism spectrum**	**Hypermobility**	**Premature (<34 Weeks)**	**Manual dexterity**	**Aiming and Catching**	**Balance**	**Total score**
**London DCD group**	17 (12/5)	9.47 (2.49)	109.4 (17.3)	90.3 (19.7)			1	1	6	1	5	1	4.24 (1.25)	5.35 (3.35)	5.0 (2.69)	3.35 (0.86)
**Brescia DCD group**	85 (73/12)	8.45 (1.89)			106.8 (15.4)	97.7 (13.7)	2	9	11	0	16	10	5.15 (1.81)	6.09 (2.57)	5.22 (2.26)	4.25 (1.67)
**Control group**	69 (35/34)	9.12 (1.81)	105.7 (10.71)				0	0	0	0	0	0	≥7	≥7	≥7	≥7

*Legend: M, male; F, female; sd, standard deviation; BPVS-II, British Picture Vocabulary Scale; CPM, Coloured Progressive Matrices; WPPSI III, Wechsler Preschool and Primary Scale of Intelligence—third version; WISC IV, Wechsler Intelligence scale for children—fourth version; ADHD, Attention Deficit Hyperactivity Disorder.*

It should be noted that a lower performance on the total impairment score than on individual subsection scores, as seen here, is a common feature of M-ABC data sets (e.g., Ashkenazi et al., 2013; Romeo et al., 2018; Ricci et al., 2021) and of the published norms, presumably reflecting the incomplete correlation of the tests in each subsection.

In London, children with DCD were recruited through advertisements placed with the Dyspraxia Foundation. All had received a diagnosis of DCD, by consultant paediatricians or occupational therapists and met DSM-5 criteria (American Psychiatric Association (APA), 2013) for DCD. Age at diagnosis of DCD ranged from 4 years 10 months to 8 years 9 months. In Brescia, all the children consecutively referred to the Unit of Child Neurology and Psychiatry of ASST Spedali Civili of Brescia between October 2016 and October 2019 for a suspected diagnosis of DCD were included in the study if they met DSM-5 criteria for a diagnosis of DCD. Age at diagnosis of DCD therefore corresponded to age of enrolling in the Brescia group.

#### Typically Developing Children (Typically Developing Control Group)

TD children in the Control group were recruited in three United Kingdom schools. Parents and guardians were invited to give consent for their children’s participation. Consent was given for 87 children, but two children were excluded due to teacher reports of a diagnosis of ADHD. Following assessment, 16 further children were excluded from the sample for performance below the 15th percentile on the M-ABC (Henderson and Sugden, 1992) or the British Picture Vocabulary Scale (BPVS-II; Dunn et al., 1997). Here we present data from 69 children aged between 5:0 and 12:11 years, to match the age range of the DCD sample. Their details are included in Table 1. However, it is not meaningful to analyse individual M-ABC scores for this TD group, since the M-ABC is scored such that performance on any item above the 25th centile receives the same score, i.e., data on a TD sample shows a very strong ceiling effect. IQ data (other than receptive vocabulary score) were also not available for the TD group.

### Procedures

#### Assessments of Motor Competency

The M-ABC was used to measure gross and fine motor competency. The London group were tested with the first version of the M-ABC (Henderson and Sugden, 1992) and the Brescia group with the M-ABC-2 (Henderson et al., 2007; Biancotto et al., 2013). Both versions comprise three subsections measuring manual dexterity, aiming and catching, and balance skills. Scores from the three subsections were weighted as specified in the test manual to produce a total impairment score. The M-ABC and M-ABC-2 use different scoring scales, but both are defined in terms of centiles within the population for the age range concerned. For the purpose of this study, both scales were converted into centiles and standard scores to allow analysis of the combined data.

#### Assessments of Intellectual Ability

The London group of children with DCD were assessed on Raven’s Coloured Progressive Matrices (Raven et al., 2008) as a test of fluid IQ, and both children with DCD and TD controls were assessed on the BPVS-II (Dunn et al., 1997). The Brescia DCD group were assessed for both verbal and non-verbal ability using the Wechsler Intelligence scales (WPPSI III = Wechsler Preschool and Primary Scale of Intelligence or WISC IV = Wechsler Intelligence Scale for Children; Wechsler, 2008, 2012). Performance on the WPSSI III or WISC IV and the CPM was used as a measure of full IQ in the present study. Results on these tests of intellectual abilities are included in Table 1 for information.

#### Assessment of Global Form and Motion Sensitivity

**FIGURE 1:**
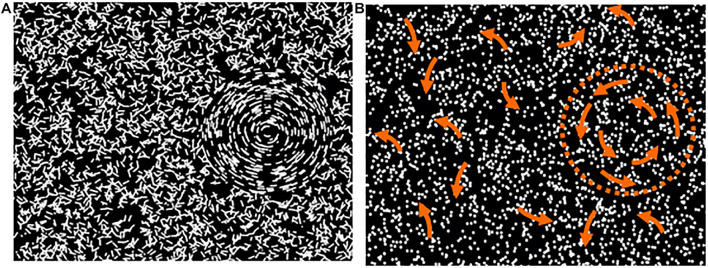
Schematic depiction of examples of the global form **(A)** and global motion **(B)** displays. The arrows in B depict the directions of motion inside and outside the marked circular region—neither the arrows nor the dotted circle were visible in the test.

Versions of the “Ball in the Grass” test developed by Dr. John Wattam-Bell in London (see Braddick et al., 2016) were used in both centres to test children’s coherence thresholds for global form (GF) and global motion (GM). Sensitivity to global form and motion was determined by the threshold for detecting global structure as a percentage of coherently organized elements embedded among random noise elements. The test used concentric stimulus displays (Atkinson and Braddick, 2005) which are designed to make the form and motion tasks as comparable as possible, in terms of cognitive demand. Children viewed a laptop computer screen and in each presentation were asked to report whether a circular region—“the ball,” containing concentrically organized short arcs (for GF) or trajectories of moving dots (for GM), was hiding in “the grass” (a background of randomly oriented arcs or randomly directed motion elements) on the left or right of centre (see Figure 1). For GM testing, the moving dots had asynchronous limited lifetimes to prevent local tracking and minimise coherent stimulus flicker.

Dimensions of the displays were slightly different for the software versions used in the two studies; details are given in Table 2.

**TABLE 2:** Parameters of the global form (GF) and global motion (GM) stimuli used in the Brescia and London studies.

**Test**	**Display size (deg arc) viewed at 50 cm**	**GM number of dots**	**GM Dot diameter (min arc)**	**GM Dot speed (deg/s)**	**GM Dot lifetime (frames, ms)**	**GF number of arcs**	**GF arc size (min)**	**Target region diameter (deg)**	**Distance target centre from screen centre (deg)**
**Brescia**	25 × 18	3,000	11	4.1	8, 133	3,000	42 × 8	9.5	6.3
**London**	32 × 24	3,000	17	4.5	8, 133	2,000	84 × 17	9.5	8.0

Each child in this study completed one run with form and one run with motion. On each trial, the structured target region was presented randomly on the left or right of centre, and the child was asked to point to the side which contained the circular pattern, or for older children to press the corresponding arrow key on the keyboard. Each run began with coherence fixed at 100% with feedback, and these trials were continued until the tester was satisfied that the child understood the task. In the following test phase, the coherence level of the target region was varied without feedback according to the PSI adaptive procedure (Kontsevich and Tyler, 1999) giving an estimate of coherence threshold after the completion of 30 trials. Most children enjoyed the “Ball in the Grass” game and completed testing without difficulty.

#### Normative Global Form and Global Motion Data

The normative data from earlier samples in London (*N* = 184, Atkinson and Braddick, 2005) and San Diego (*N* = 153, Braddick et al., 2016) were used to derive percentile values and hence scaled scores for GF and GM sensitivity within each 1-year age band in the range of the present samples, with 41–65 children contributing data to each age band. The London sample was tested with one determination of each threshold whereas the San Diego data were based on the mean of two determinations of each threshold for each child. These scaled scores were used in the analyses below of GF and GM sensitivity in relation to M-ABC scores.

**FIGURE 2:**
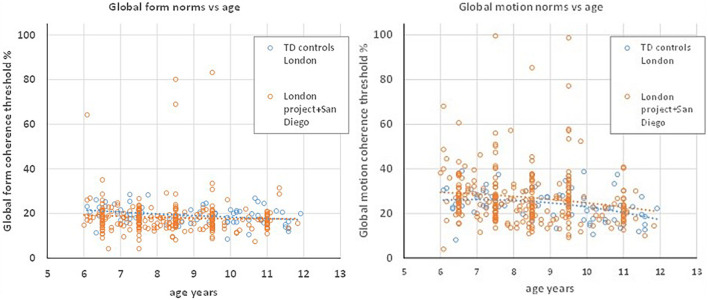
Global form **(left)** and global motion **(right)** thresholds as a function of age for typically developing children tested on the “Ball in the Grass” test in the London control group reported here (blue) and the combined earlier groups from London and San Diego (orange). The latter combined group is the source of the norms used to calculate standard scores in the analysis of DCD children. The coloured dotted lines are quadratic fits to the respective data sets and are closely aligned for the two samples.

The test stimuli used in these normative studies were identical to those used in the Brescia DCD group of the present study. The TD control group was tested in London with the stimulus dimensions as for the London DCD group, i.e., those given in Table 2. Figure 2 plots normative data from the combined London and San Diego samples, and from the present TD control group, showing that the functions relating global form and motion sensitivity to age are aligned for these groups despite the small stimulus differences. A regression analysis (see section “*Statistical Analysis”* below) showed no significant effect of group, or interaction of group with age for either GM or GF. It was therefore considered appropriate to use the London TD group as controls for both the London and Brescia DCD groups, and the earlier data set as a basis for the scaled scores.

#### Statistical Analysis

All analyses were performed using R (version 4.0.3; R Core Team, 2016). Continuous data (GM, GF and M-ABC scores) were modelled using ordinary least square regression models. Non-linear trends were modelled using restricted cubic splines with 3 knots. Estimates are reported with associated 95% confidence intervals. All tests were two-sided and assumed a significance level of 5%.

## Results

### Comparison of Developmental Coordination Disorder and Typically Developing Control Groups

Table 1 shows the descriptive statistics of the DCD group (London Group plus Brescia Group) and the TD control group. We first compared scaled scores for global motion and global form coherence sensitivity between the DCD and TD Control groups in order to examine whether children with DCD showed evidence of dorsal stream vulnerability. Neither age nor gender made a significant contribution to these scores for the two groups together, either on GM (age: *p* = 0.441; gender: *p* = 0.805) or on GF (age: *p* = 0.976; gender: *p* = 0.382).

**FIGURE 3:**
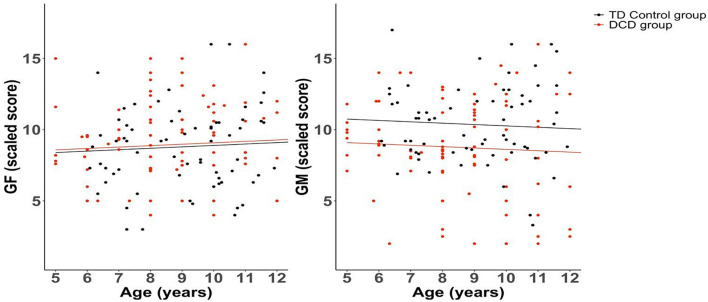
Linear regression models for global form (GF) and global motion (GM) scaled scores as a function of age (years), separately for DCD and TD control groups.

**FIGURE 4:**
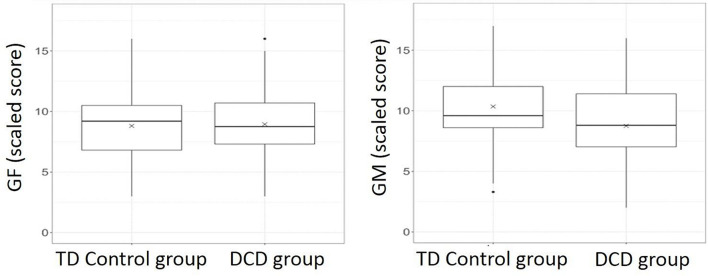
Box-and-whisker plots showing medians and interquartile range for global form (GF) and global motion (GM) scaled scores separately for DCD and TD Control groups. In each group, horizontal line = median; box = interquartile range (IQR); vertical whiskers extend to extreme values that are not more than 1.5*IQR away from the box. The adjusted mean given in the text for each group is indicated by X on each plot.

Compared to the control group, the DCD group showed significantly poorer scaled scores for coherence sensitivity to global motion (“least squares” means adjusted for age and gender in the model: 8.68 vs. 10.38, delta 1.7, CI_95__%_ 0.64; 2.75, *p* = 0.002), but not to global form (“least squares” means: 8.78 vs. 8.97, delta −0.19, CI_95__%_ −1.14; 0.77, *p* = 0.695). Figure 3 shows the individual data and linear relationships fitted from the model for each group, and Figure 4 presents box-and-whisker plots showing the median, quartile and range for each of these data sets.

In the DCD group, full scale IQ (FIQ) was not associated with global motion coherence sensitivity (*p* = 0.39) while better global form coherence sensitivity was associated with a higher FIQ (*p* = 0.006). This pattern was also replicated in the results from the WISC IV (Brescia DCD group only; GM: *r* = 0.17, *p* = 0.23 and GF: *r* = 0.28, *p* = 0.02). Overall, global form and global motion were significantly correlated (*r* = 0.29, *df* = 169, *p* < 0.001), as previously reported by Braddick et al. (2016).

### Relationship of Movement-ABC Scores to Global Form and Motion in the Developmental Coordination Disorder Group

**FIGURE 5:**
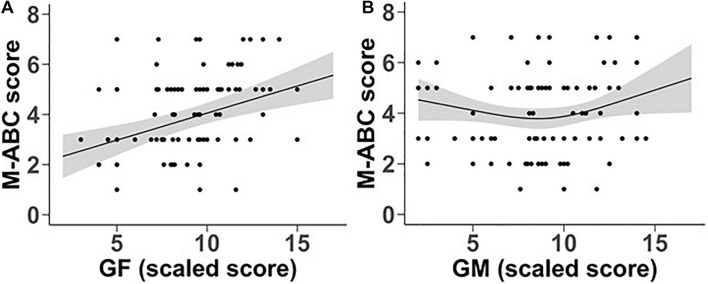
Relationship between M-ABC scores and scaled scores for global form **(A)** and global motion **(B)** coherence sensitivity. Lines represent estimated mean values while shaded grey bands represent 95% confidence intervals.

In order to examine the relationship of GM and GF sensitivity to the level of motor deficit of individuals within the DCD group, the association of GM and GF scaled scores to the total M-ABC standard scores was analysed, in a regression model including FIQ scores and age. The same analysis was carried out with each of the subsection scores (Manual Dexterity, Aiming and Catching, and Balance). Figure 5 shows the relationship between total M-ABC scores and GF and GM scaled scores. Total M-ABC standard scores showed a significant linear relationship with global form coherence sensitivity (*p* < 0.001).

The significant quadratic relationship with global motion coherence sensitivity (*p* = 0.046) was more complex. As shown in Figure 5B, low values of GM scaled scores are unrelated to M-ABC, but M-ABC increases over the high range of GM scaled scores. This relationship is not age-dependent (*p* = 0.329) and is not influenced by full IQ test scores (*p* = 0.326).

Examination of scores for each M-ABC subsection showed that global form coherence sensitivity was significantly linearly related to Aiming and Catching (*p* = 0.003) and Balance (*p* = 0.001), with the relationship between Manual Dexterity and global form coherence sensitivity only marginally significant (*p* = 0.07). There was no significant linear relationship between scores for each M-ABC subsection and global motion coherence sensitivity (Manual Dexterity *p* = 0.370; Aiming and Catching *p* = 0.639; Balance *p* = 0.987).

## Discussion

As discussed in the Introduction, previous studies comparing children with DCD to TD controls yielded conflicting results. The present study used a much larger sample of children, uniformly confirmed as having DCD both by DSM-5 criteria and by results on a standardized battery (M-ABC) and allowed variation of global form and motion sensitivity with age to be taken into account. The contrast between the sensitivity to global form and motion, from two parts of the “Ball in the Grass” test designed to be closely similar in their general cognitive demands, provides some confidence that the difference between children with DCD and TD controls is not a consequence of any general difference between the groups.

However, the results within the DCD group indicate that the relationship between motor skill deficits and global visual perception is more complex than indicated from the comparison with TD controls. In children with DCD, with varying levels of motor skill deficits, motor performance as assessed by the M-ABC showed a significant linear association with global form sensitivity even when age and IQ effects were taken out in the regression model. Global motion sensitivity showed no such linear relationship. However, the significant quadratic relationship seen in Figure 5 indicates that over the lower range of global motion sensitivity, M-ABC scores showed no systematic relationship with global motion sensitivity, but that at median levels of global motion sensitivity and above, higher motion sensitivity was associated with higher levels of motor skill.

Thus, in response to the questions posed in the Introduction, we find (a) that motor skill performance is related to both global form and global motion sensitivity, and this relationship is unrelated to either (b) age or (c) IQ measures. It should be noted that sensitivity measures for global motion and global form are significantly correlated with each other. However, the relationships to motor skill illustrated in Figure 5 come from a regression model in which both form sensitivity and motion sensitivity are entered. Therefore, the distinctive patterns of these relationships to motor skill must reflect the contribution of parts of the variance that are unique to global form and global motion sensitivity, respectively. Neither relationship shows a variation with age, and the results of including IQ in the regression model indicate that the associations do not simply reflect general cognitive ability.

A possible interpretation of these results is that two separate visual components are linked to Developmental Coordination Disorder. The first of these visual components is associated with global form sensitivity and acts uniformly on the range of motor impairment seen in DCD but has little impact over the higher range of motor performance which differentiates typically developing controls from children diagnosed with DCD. It should be noted that global form sensitivity also showed a linear association with IQ in this population.

A second visual component, associated with global motion sensitivity, only starts to contribute when it reaches a relatively high level in this cohort, where it begins to be associated with higher levels of motor skills. This second component is the dominant factor associated with the much higher levels of motor skill performance which differentiate typically developing controls from children diagnosed with DCD.

Global motion is processed in brain areas within dorsal stream networks such as the intraparietal sulcus (Sunaert et al., 1999; Braddick et al., 2001; Helfrich et al., 2013) and has been taken as a functional signature of the dorsal cortical stream. The dorsal stream is known to be important for sensory-motor transformations (Buneo and Andersen, 2006; Kravitz et al., 2011). These sensory-motor transformations are essential for the motor skills tested by the M-ABC: spatial vision is required for fine manual control tasks such as bead threading and placing pegs in holes; visual motion processing is key for ball skills (Regan, 1997) and dynamic balance (Sparto et al., 2006). The relationship of these skills to global motion processing is therefore likely to reflect a shared basis in the structure and function in the dorsal cortical stream, and its vulnerability to adverse conditions in development. However, it is only the higher levels of motion sensitivity which reflect this shared basis; when global motion sensitivity is at the lowest levels, other factors appear to dominate in determining the level of motor skill.

A neural interpretation of the linear relationship of global form sensitivity with the lower range of motor abilities within the DCD group is less clear. Low levels of motor skills appear to be related to form sensitivity, but at the levels which differentiate typical development from DCD, form sensitivity shows no association with motor skills. Further research and analysis may yield more insight into what aspects of children’s motor coordination are linked to visual dorsal and ventral stream performance, respectively. It should also be noted that dorsal and ventral processing are not independent of each other; the two streams are linked by the vertical occipital fasciculus (Yeatman et al., 2014) and in other connections. The role of these connections in the development of skilled motor behaviour in children is yet to be explored.

### Limitations

It was not possible to examine the relationship between motor performance and global form and motion sensitivity in the typically developing control group, since the M-ABC is scored to differentiate between children’s motor skills within the low end of the performance range and therefore will not represent variations in motor skills in the typical control group. The presented data are derived from two samples which differed in their recruitment method, which may be responsible for the difference in overall level of impairment apparent in Table 1; it is plausible that the London children whose families participated in the Dyspraxia Foundation, and who were overall somewhat older, had more severe impairment than the younger, suspected cases who formed the Italian sample. There were also small differences in stimulus parameters as described above and the version of the M-ABC battery used. However, (a) the stimulus differences do not appear to lead to any systematic differences in performance of typically developing children (Figure 2); (b) the differences between M-ABC and M-ABC-2 are primarily in the scoring system, which have been handled here by deriving centiles and hence standard scores from each version of the battery, allowing a unified data presentation. Both samples met the same criteria in terms of their M-ABC performance, and their inclusion in a common analysis adds generality and strength to our conclusions.

## Conclusion

The present study of a large, well-characterised group of children diagnosed with Developmental Coordination Disorder shows that the presence and level of their deficit in motor skills have clear associations with global visual coherence sensitivity to both static form and motion. The deficit in global motion sensitivity is shared with a range of other neurodevelopmental disorders and is expected from the known sensory-motor functions of networks in the dorsal cortical stream. However, when a marked deficit of global form processing is present, this dominates the association between visual perceptual and motor skill impairments. This latter relationship will hopefully be pursued further in future research at both neural and functional levels to enable effective interventions.

## Data Availability Statement

The raw data supporting the conclusions of this article will be made available by the authors, without undue reservation.

## Ethics Statement

The studies involving human participants were reviewed and approved by the Comitato Etico di Brescia, Brescia, Italy (NP 3513) and UCL Ethics Committee, London, United Kingdom (2807/002). Written informed consent to participate in this study was provided by the participants’ legal guardian/next of kin.

## Author Contributions

SM and FC: study design and conception, data collection, data interpretation, manuscript writing, and critical revision of the article. JA and OB: design and provision of test material, advice on study design and interpretation, part drafting, and critical revision of manuscript. PM and JG: data collection and critical revision of the article. SC: data analysis and interpretation, critical revision of the article. EF: study design and conception, data interpretation, and critical revision of the article. All authors provided final approval of the version to be published.

## Conflict of Interest

The authors declare that the research was conducted in the absence of any commercial or financial relationships that could be construed as a potential conflict of interest.

## Publisher’s Note

All claims expressed in this article are solely those of the authors and do not necessarily represent those of their affiliated organizations, or those of the publisher, the editors and the reviewers. Any product that may be evaluated in this article, or claim that may be made by its manufacturer, is not guaranteed or endorsed by the publisher.
